# Optimal glycated hemoglobin A1c value for prediabetes and diabetes in patients with pancreatic diseases

**DOI:** 10.3389/fendo.2023.1208187

**Published:** 2023-07-06

**Authors:** Guanhua Chen, Rui Zhang, Chunlu Tan, Xubao Liu, Lei Yu, Yonghua Chen

**Affiliations:** ^1^ Division of Pancreatic Surgery, Department of General Surgery, West China Hospital, Sichuan University, Chengdu, China; ^2^ Department of Pharmacy, West China Hospital, Sichuan University, Chengdu, China

**Keywords:** diabetes mellitus, prediabetes, oral glucose tolerance test, glycated hemoglobin A1c, pancreatic disease

## Abstract

**Background:**

Some articles suggest that using HbA1c alone for diabetes diagnosis is inappropriate. It requires considerable researches to explore the efficacy of HbA1c for diagnosing hyperglycemia in patients with pancreatic disease.

**Methods:**

This study analyzed 732 patients, comprising of 331 without pancreatic disease and 401 patients diagnosed with pancreatic diseases. All participants underwent the HbA1c assay and oral glucose tolerance test. Kappa coefficients were calculated to assess agreement between the HbA1c and glucose criteria. The receiver operating characteristic curve (ROC) was used to calculate the optimal HbA1c value. DeLong test was analyzed to compared the aera under curves (AUCs).

**Results:**

There were 203 (61.3%) patients with NGT, 78 (23.6%) with prediabetes, and 50 (15.1%) with diabetes in patients without pancreatic diseases. In patients with pancreatic disease, 106 participants were diagnosed with NGT (36.4%), 125 with prediabetes (31.2%), and 130 with diabetes (32.4%). Patients with pancreatic disease exhibited elevated levels of bilirubin, transaminase enzymes, aspartate transaminase, high density lipoprotein cholesterol and total bile acid. The sensitivity and specificity of the HbA1c (6.5%) for diagnosing pancreatic diabetes were 60.8% (95% CI 52.3, 69.3) and 92.6% (95% CI 89.5, 95.7). In prediabetes, the sensitivity and specificity of HbA1c (5.7%) is 53.2% (44.3, 62.0) and 59.6 (51.5, 67.6). The optimal HbA1c value for diagnosing diabetes was 6.0% (AUC = 0.876, 95% CI 0.839, 0.906), with the sensitivity of 83.8% and the specificity of 76.8%. The optimal HbA1c value for the diagnosis of prediabetes was 5.8% (AUC = 0.617, 95% CI: 0.556, 0.675), with the corresponding sensitivity and specificity of 48.0% and 72.6% respectively. The combined tests (HbA1c, 6.0% or FPG, 7.0mmol/L) presented the sensitivity of 85.7% (95% CI 79.1, 91.3)and the specificity of 92.6% (95% CI 87.6, 97.3) in pancreatic diabetes.

**Conclusion:**

From our results, the recommended HbA1c by ADA criterion may not be sufficiently sensitive to diagnose hyperglycemia in pancreatic disease. The optimal value of 5.8% and 6.0% improved the accuracy for diagnosing prediabetes and diabetes and should be considered to be applied. Besides, we advocate the combination of HbA1c and FPG test for the diagnosis of diabetes in patients with pancreatic diseases.

## 1 Introduction

Pancreatic (type 3c) diabetes mellitus (T3cDM) occurs due to inherited or acquired pancreatic disease or pancreatectomy (1) and accounts for 5–10% of patients with diabetes in Western countries (2). Different from type 1 and type 2 diabetes (T2DM), T3cDM has a unique pattern of metabolic and hormonal characteristics and a high incidence among patients with pancreatic disease (3). Reduced β cell functions due to abnormal inflammation response, lacked related endocrine peptide, and malnutrition were considered as possible mechanisms in T3cDM (4). In a prospective study by Rahul Pannala et. al, approximately 50% pancreatic ductal adenocarcinoma (PDAC) patients had diabetes mellitus and approximately 85% of them had elevated fasting plasma glucose (FPG) levels at diagnosis (5, 6). Another research showed that 30%-40% of patients with chronic pancreatitis (CP) were found to have diabetes (7). These studies highlight the condition that glucose homeostasis disturbance may be a near-universal phenomenon in pancreatic disease.

The method used to identify diabetes status, as recommended by the American Diabetes Association’s (ADA) criteria, also applies to glucose metabolism disorder among pancreatic disease patients, which contains glucose criteria (FPG, and 2-hour plasma glucose during the oral glucose tolerance test, 2h OGTT) and hemoglobin A1c (HbA1c), The OGTT is always considered as the gold standard; however, it is infrequently applied due to its limitations of being time-consuming and cumbersome. Due to its convenience and stability, HbA1c has evolved into an alternate tool to OGTT (8). The ADA recommends using HbA1c levels of 5.7% or 6.5% to diagnose prediabetes or diabetes due to its association with retinopathy (9, 10). However, variables such as age, ethnicity, and specific clinical conditions that affect hemoglobin glycation rates may impact the accuracy of HbA1c testing (11–14). Recently, in clinical settings involving patients with obesity, high-altitude polycythemia, liver disease, acute hemorrhage or blood transfusion, numerous articles have indicated that the commonly utilized HbA1c value of 6.5% may result in either underdiagnosis or overdiagnosis of diabetes mellitus (13, 15, 16). Diabetes secondary to pancreatic disease is different from conventional diabetes, which leads us to consider whether the diagnosis by HbA1c alone is appropriate. Elevated bilirubin, poor nutritional status, inflammation and impaired liver function - all common in pancreatic disease - have been demonstrated to impact HbA1c levels in the body (17–19). Thus, we conducted a cross-sectional study to explore the performance of HbA1c for diagnosing prediabetes and diabetes secondary to pancreatic diseases and analyzed the optimal threshold of HbA1c level through ROC curve. Furthermore, we try to analyze the performance of combination of FPG (7.0mmol/L) and HbA1c for diagnosing diabetes in our study.

## 2 Methods

### 2.1 Study design and population

**Figure 1:**
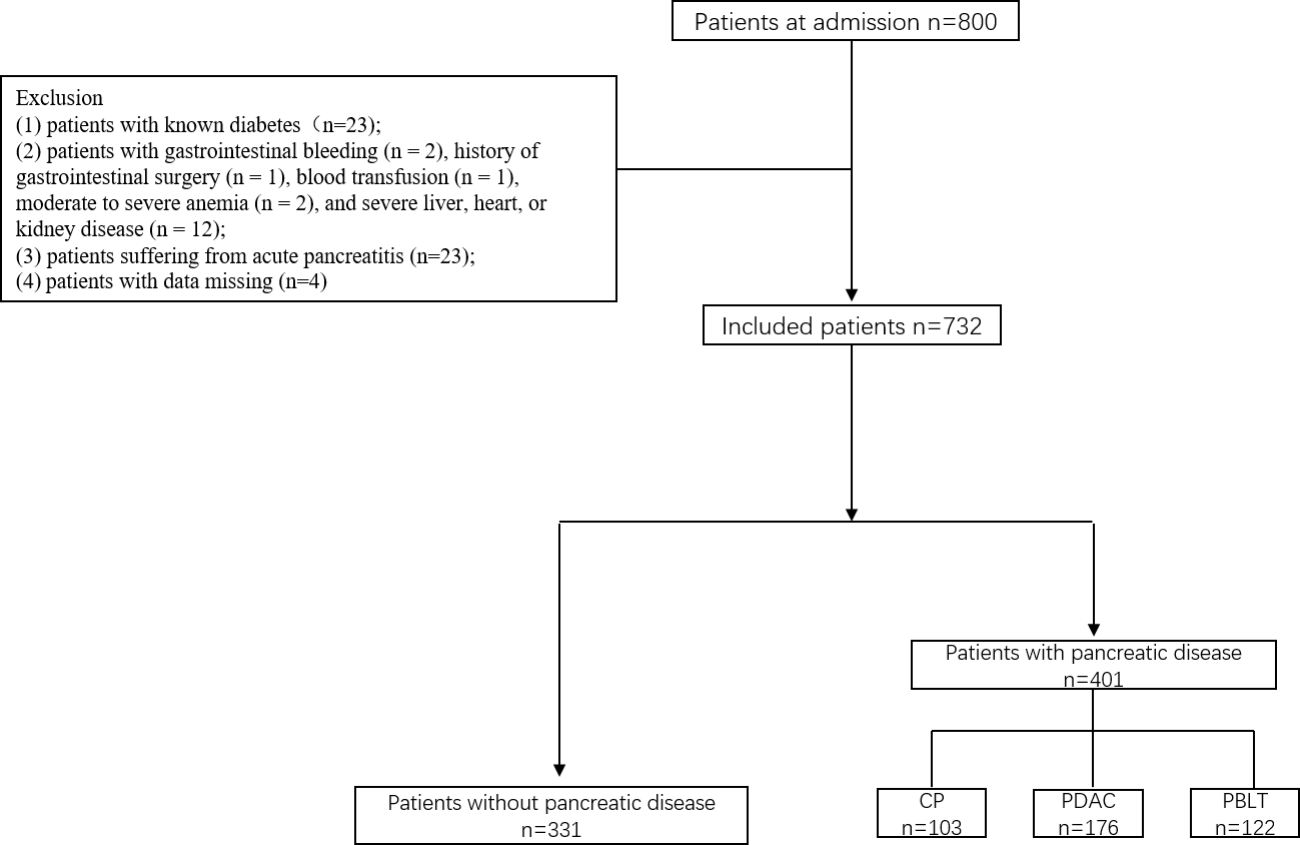
Flow diagram of the recruitment of participants.

This was a cross-sectional study conducted at the Department of Pancreatic Surgery, West China Hospital. During the research, all procedures abided by the guidelines of the ethical standards of the institutional research committees of the West China Hospital and the Helsinki Declaration of 1964 and its later amendments or comparable ethical standards. Patients in West China Hospital of Sichuan University, between June 2017 and December 2019 were enrolled in our study. They were divided into two groups: patients without pancreatic diseases and patients with pancreatic disease including chronic pancreatitis, pancreatic ductal adenocarcinoma and pancreatic benign and low-grade tumor. The inclusion criteria were as follows (1): patients aged 18 or older; (2) current lifestyle measures, including medicine and physical activity, that did not influence the results of tests (excessive dieting, alcohol use, glucocorticoids, salicylic acid, β-blockers or other medications that may interfere with blood sugar testing; in our study, no people used those medications or food); and (3) patients provided informed consent. The exclusion criteria were as follows: (1) patients with known diabetes(n=23); (2) patients with gastrointestinal bleeding (n = 2), history of gastrointestinal surgery (n = 1), blood transfusion (n = 1), moderate to severe anemia (n = 2), and severe liver, heart, or kidney disease (n = 12); (3) patients suffering from acute pancreatitis (n=23); (4) patients with data missing (n=4) (**Figure 1**).

### 2.2 Outcomes and data collection

Our primary outcome was the differential performance of HbA1c for detecting prediabetes or new-onset diabetes compared to ADA’s glucose criteria. HbA1c was tested by high-performance liquid chromatography (G7 HbA1c Standard Analysis Mode (1.2 min) HLC-723G7, TOSOH Japan) at admission according to the method certified by the National Glycohemoglobin Standardization Program (NGSP). After at least an 8-hour overnight fast, the FPG and OGTT was performed. All patients underwent the glucose test with a 3-day diet containing 150 g carbohydrate per day to avoid hunger diabetes. FPG levels were tested using the glucose oxidase method (a cobas8000 analyzer, Roche Diagnostics, Basel, Switzerland). In addition, on the same day as the OGTT, 5ml of venous blood was drawn for testing liver function (total bilirubin, alanine transaminase, aspartate transaminase albumin, and total bile), creatinine, and lipid metabolism (triglyceride, high density lipoprotein cholesterol, low density lipoprotein cholesterol, and cholesterol levels).

### 2.3 Definition

According to glucose criteria by the ADA, patients with FPG ≥ 7.0 mmol/L or/and 2h OGTT ≥ 11.1 mmol/l were diagnosed with diabetes mellitus. Prediabetes was defined as FPG of 5.6 to 6.9 mmol/L and 2h OGTT of 7.8 to 11.0 mmol/L. FPG < 5.6 mmol/L, and 2h OGTT < 7.8 was considered as normal glucose tolerance (NGT).

### 2.4 Statistical analysis

Data are presented as the frequencies for categorical variables and the means ± standard deviation (SD) for continuous variables. All data were analyzed by SPSS version 24.0 (IBM, New York, US). Categorical data were analyzed using Pearson’s chi-square test or Fisher test. Continuous data were analyzed using samples t test or Kruskal–Wallis H nonparametric test. Kappa coefficients were calculated to assess agreement between glucose test results obtained from the HbA1c and glucose criteria criteria. The negative predictive value (NPV) and positive predictive value (PPV) were calculated as described previously (20). The receiver operating characteristic curve (ROC) was used to calculate the area under the curve (AUC) of HbA1c to diagnose diabetes and prediabetes. The optimal HbA1c cutoff value was generated according to the Youden Index to determine the maximum potential effectiveness while balancing sensitivity and specificity. DeLong test was analyzed to compared the AUCs. A two-sided P value less than 0.05 was considered statistically significant.

## 3 Results

### 3.1 Characteristic of patients

We conducted an analysis on a total of 732 patients, comprising of 331 individuals without pancreatic disease and 401 patients diagnosed with pancreatic diseases. In the pancreatic disease group, there were 176 (43.9%) patients with pancreatic ductal adenocarcinoma (PDAC), 103 (25.7%) patients with chronic pancreatitis (CP) and 122 (30.4%) patients with pancreatic benign and low-grade tumor (PBLT) including 37 non-function pancreatic neuroendocrine tumor, 38 intraductal papillary mucous neoplasm, 37 solid pseudopapilloma, and 20 cystadenoma. **Table 1** presents the important characteristic of patients. The mean age was 50.17±14.38 in patients without pancreatic diseases and 55.0% of them were males. In patients with pancreatic disease, the mean age was 54.67 ± 13.30 years, and 56% of them were male. According to ADA’s glucose criteria, there were 203 (61.3%) patients with NGT, 78 (23.6%) with prediabetes, and 50 (15.1%) with diabetes in patients without pancreatic diseases. In patients with pancreatic disease, 146 participants were diagnosed with NGT (36.4%), 125 with prediabetes (31.2%), and 130 with diabetes (32.4%). In our study, patients with pancreatic disease exhibited a higher prevalence of diabetes (32.4% vs 15.1% P<0.001). A significant correlation exists between pancreatic disease and diabetes (**Sup 1**)

**Table 1:** Characteristics of patients.

	Patients without pancreatic disease (n=331)	Patients with pancreatic disease (n=401)			
		All (401)	CP(n=103)	PDAC(n=176)	PBLT (n=122)
Age (year)	50.17±14.38	54.67±13.30^***^	50.10±13.91	59.80±11.07^***^	51.32±13.75
Male Sex (n, %)	182 (55.0)	225(56.1)	58 (56.3)	99 (56.2)	68 (55.7)
Body mass index (kg/m2)	24.41±4.28	22.34±3.40	21.60±3.25	22.20±3.30	23.13±3.50
Systolic blood pressure (mmHg)	123.38±18.55	123.97±16.48	123.11±17.39	124.73±15.59	123.64±17.10
Diastolic blood pressure (mmHg)	77.49±11.58	78.27±10.62	77.39±10.60	78.45±10.71	78.68±10.58
Amylase (U/L)	49.51±40.36	103.44±122.03^***^	121.70±135.63^***^	103.42±105.98^***^	87.83±130.72^***^
HbA1c (%)	5.9±1.53	6.3±1.61^**^	6.4±1.63***	6.6±1.64^***^	6.3±1.45**
Fasting plasma glucose (mmol/L)	6.21±1.88	6.04±2.17	5.92±2.08	6.53±2.40	5.45±1.70
2h OGTT (mmol/L)	8.79±4.47	10.30±5.16^***^	10.21±5.34***	11.66±5.25***	8.41±4.23
Diabetes status (n, %)					
NGT	203 (61.3)	146 (36.5)^***^	50 (48.6)^***^	36 (26.5)^***^	69 (56.6)^**^
Prediabetes	78 (23.6)	125 (31.1)	26 (25.2)	59 (33.5)	31 (25.4)
Diabetes	50 (15.1)	130 (32.4)	27 (26.2)	81 (46.0)	22 (18.0)
Total bilirubin (umol/L)	9.18±6.48	36.52±77.30^***^	19.28±54.16^***^	61.94±101.84^***^	14.45±25.53^***^
Alanine transaminase (IU/L)	18.28±10.78	36.42±43.63^***^	42.97±69.98^***^	60.95±90.30^***^	24.16±34.44^***^
Aspartate transaminase (IU/L)	23.17±12.16	47.36±33.49^***^	56.38±27.68^***^	69.44±70.77^***^	31.19±11.89^**^
Albumin (g/L)	45.58±6.53	42.69±4.56^*^	43.03±4.05^*^	41.80±5.02^***^	44.66±4.04
Creatinine (umol/L)	63.56±14.46	65.56±17.12	68.41±21.34	64.97±15.09	64.02±15.75
Triglyceride (mmol/L)	1.37±1.02	1.53±1.04	1.75±1.43^**^	1.57±0.96	1.30±0.63
Cholesterol (mmol/L)	4.11±1.33	4.39±1.29	4.47±1.18^*^	4.45±1.50	4.29±0.93
High density lipoprotein cholesterol (mmol/L)	1.37±0.57	1.12±0.48^*^	1.13±0.47^**^	1.03±0.54^**^	1.24±0.38
Low density lipoprotein cholesterol (mmol/L)	2.53±1.01	2.46±0.90	2.55±0.92	2.35±0.95	2.54±0.81
Total bile acid (umol/L)	6.16±12.26	19.22±47.47^***^	12.27±28.56^***^	32.22±65.43^***^	6.37±10.80
Hemoglobin (g/L)	132.29±17.47	130.72±16.44	131.79±19.39	128.18±15.93^*^	133.39±13.86

All compared to patients without pancreatic disease: * P<0.05; ** P<0.01; *** P<0.0001.

PDAC, pancreatic ductal adenocarcinoma; CP, chronic pancreatitis; PBLT, pancreatic benign and low-grade tumors; NGT, Normal glucose tolerance; 2h OGTT, 2-hour plasma glucose during the oral glucose tolerance test.

### 3.2 Agreement between HbA1c and glucose criteria

**Table 2** presents the agreement of prediabetes and diabetes according to HbA1c and glucose criteria by ADA. The Kappa coefficients was 0.653 (0.597, 0.714) and 0.310 (0.208, 0.379) in patients without pancreatic diseases. In our study, 130 patients were diagnosed as diabetes by ADA’s glucose criteria and 99 were diagnosed with diabetes by the ADA’s HbA1c criteria. However, only 79 (60.8%) were identified as diabetes by both. Besides, 51 (39.2%) patients were underdiagnosed by the proposed HbA1c and 20 patients were overdiagnosed as diabetes by HbA1c. As for prediabetes, only 66 (52.8%) patients were diagnosed by both criteria, indicating that nearly half patients with prediabetes may not be detected accurately by HbA1c alone. Similarly, in patients with different pancreatic disease, there is an apparent discordance in HbA1c for diagnosing diabetes and prediabetes, which lead us to doubt the accuracy of HbA1c applied to patients with pancreatic disease. Sensitivity, specificity, PPV and NPV of HbA1c or FPG recommended by ADA criteria were shown in **Table 3**. The diagnostic sensitivity of HbA1c (6.5% or 5.7%) for prediabetes or diabetes in our study is generally low.

**Table 2:** Agreement between HbA1c and glucose criteria by ADA.

	HbA1c			Total	Kappa coefficient (95% CI)
Glucose	<5.7	5.7~6.4	>6.5		
Patients without pancreatic disease					
NGT	171 (84.7)	16 (7.9)	6 (7.4)	202	
Prediabetes	17 (21.8)	55 (70.5)	6 (7.7)	78	
Diabetes	3 (6.0)	4 (8.0)	43 (86.0)	50	0.653 (0.597, 0.714)
Patients with pancreatic diseases					
Overall					
NGT	81 (55.5)	59 (40.9)	6 (4.1)	146	
Prediabetes	45 (36)	66 (52.8)	14 (11.2)	125	
Diabetes	8 (6.2)	43 (33.1)	79 (60.8)	130	0.310 (0.208, 0.379)
CP					
NGT	21 (51.2)	18 (43.9)	2 (4.9)	41	
Prediabetes	9 (25.7)	19 (54.3)	7 (20)	35	
Diabetes	0 (0)	9 (33.3)	18 (66.7)	27	0.343 (0.277, 0.393)
PDAC					
NGT	20 (55.6)	13 (36.1)	3 (8.3)	36	
Prediabetes	25 (42.4)	30 (50.8)	4 (6.8)	59	
Diabetes	7 (8.6)	25 (30.9)	49 (60.5)	81	0.322 (0.216, 0.368)
PBLT					
NGT	40 (58)	28 (40.6)	1 (1.4)	69	
Prediabetes	11 (35.5)	17 (54.8)	3 (9.7)	31	
Diabetes	1 (4.5)	9 (40.9)	12 (54.5)	22	0.297 (0.212, 0.365)

PDAC, pancreatic ductal adenocarcinoma; CP, chronic pancreatitis; PBLT, pancreatic benign and low-grade tumors; NGT, Normal glucose tolerance.

The glucose criteria include the fasting plasma glucose or 2h OGTT.

**Table 3:** Performance of HbA1c or fasting plasma glucose recommended by ADA in the diagnosis of diabetes and pre-diabetes among patients with pancreatic diseases.

	HbA1c or FPG	Sensitivity (%)	Specificity (%)	Positive predictive value	Negative predictive value
Diabetes	HbA1c≥6.5%				
Overall		60.8 (52.1, 69.1)	92.6 (89.5, 95.7)	79.8 (71.7, 87.8)	83.1 (78.9, 87.4)
PDAC		60.5 (49.6, 71.4)	92.6 (87.3, 98.0)	87.5 (78.6, 96.4)	73.3 (65.3, 81.4)
CP		66.7 (47.7, 85.7)	88.2 (80.7, 95.6)	55.0 (31.1, 78.9)	88.4 (81.2, 95.7)
PBLT		54.6 (31.9, 77.1)	96.0 (92.1, 99.9)	73.3 (48.0, 98.7)	90.6 (84.9, 96.2)
Prediabetes	HbA1c ≥5.7%				
Overall		53.2 (44.3, 62.0)	59.6 (51.5, 67.6)	53.2 (44.3, 62.0)	59.6 (51.5, 67.6)
PDAC		42.9 (28.5, 57.2)	63.9(47.4, 80.4)	61.8 (44.5, 79.0)	45.1 (33.0, 59.2)
CP		54.3 (36.9, 71.6)	56.1 (40.2, 72.0)	51.4 (34.4, 68.2)	58.9 (42.8, 75.1)
PBLT		54.8 (36.3, 73.4)	59.4 (47.5, 71.3)	37.8 (23.0, 52.5)	74.6 (62.7, 86.4)
Diabetes	FPG>7.0 mmol/L				
Overall		50.8 (31.4, 67.6)	95.9 (91.3, 99.9)	85.7 (73.6, 94.4)	80.3 (71.8, 86.7)
PDAC		56.8 (39.5, 72.6)	93.1 (91.3, 97.5)	82.4 (74.6, 89.3)	74.8 (53.6, 84.3)
CP		61.5 (42.5, 77.5)	98.7 (93.0, 99.3)	94.1 (87.6, 97.8)	88.4 (72.4, 90.3)
PBLT		35.0 (27.1, 46.4)	97.1 (85.3, 99.1)	82.4 (72.4, 88.6)	88.4 (80.4, 93.1)
Prediabetes	FPG>5.6 mmol/L				
Overall		42.0 (27.0, 50.3)	80.8 (70.4, 87.5)	41.1 (26.3, 56.7)	71.7 (44.8, 96.6)
PDAC		34.4 (24.5, 38.3)	79.5 (70.2, 84.5)	48.9 (40.6, 52.3)	67.9 (53.3, 73.2)
CP		14.7 (6.8, 19.2)	79.7 (69.9, 89.3)	26.1 (14.7, 32.2)	65.5 (59.3, 59.8)
PBLT		31.0 (26.5, 39.7)	79.6 (69.8, 82.5)	32.1 (27.9, 48.1)	78.7 (71.6, 87.5)

Values in table are presented as percentage with the 95% confidence interval in parenthesis.

FPG, fasting plasma-glucose; PDAC, pancreatic ductal adenocarcinoma; CP, chronic pancreatitis; PBLT, pancreatic benign and low-grade tumors; FPG, fasting plasma glucose.

### 3.3 Optimal value of HbA1c for prediabetes and diabetes

Receiver operator characteristic (ROC) curves were generated to determine optimal cutoff points detecting diabetes or prediabetes (**Table 4**). The optimal HbA1c cutoff value for diagnosing diabetes was 6.0% (AUC = 0.876, 95% CI 0.839, 0.906), and the sensitivity increased to 83.8% (95% CI 72.4, 88.7) with a corresponding specificity of 76.8% (95% CI 68.2, 81.3). The optimal HbA1c value for the identification of prediabetes was 5.8% (AUC = 0.617, 95% CI: 0.556, 0.675, P <0.001), with the corresponding sensitivity and specificity of 48.0% (95% CI 38.3, 60.6) and 72.6% (95% CI 61.3, 81.9) respectively. Furthermore, we analyzed the optimal cutoff points in different types of pancreatic diseases. The optimal HbA1c threshold values for diabetes secondary to pancreatic diseases were 6.2% (PDAC), 6.0% (CP) and 6.0% (PBLT) with the corresponding AUC of 0.870 (95% CI 0.811, 0.916), 0.897 (95% CI 0.822, 0.948) and 0.893 (95% CI 0.824, 0.942), respectively. In prediabetes, the optimal cutoffs for HbA1c were 5.8%, 5.9% and 5.6, with corresponding AUCs of 0.55 (95% CI 0.445, 0.652), 0.688 (95% CI 0.572, 0.790) and 0.610 (95% CI 0.507, 0.706), respectively.

**Table 4:** Stratified analysis of optimal HbA1c for diagnosing diabetes and prediabetes in patients with pancreatic diseases.

	Optimal HbA1C cutoff points	Sensitivity (%)	Specificity (%)	Positive predictive value (%)	Negative predictive value (%)	AUC (95%)	P
Diabetes (all 130)	6.0	83.8 (72.4, 88.7)	76.8 (68.2, 81.3)	74.7 (70.7, 83.8)	84.8 (71.2, 90.1)	0.876 (0.839, 0.906)	<0.001
Disease							
PDAC (81)	6.2	72.8 (63.3, 77.5)	86.3 (79.4, 88.8)	77.0 (65.2, 81.3)	82.4 (79.5, 87.2)	0.870 (0.811, 0.916)	<0.001
CP (27)	6.0	92.6 (86.3, 97.4)	68.4 (57.2, 72.8)	56.8 (47.3, 62.6)	92.4 (83.8, 96.4)	0.897 (0.822, 0.948)	<0.001
PBLT (22)	6.0	81.8 (72.3, 88.4)	87.0 (78.2, 91.2)	51.6 (38.8, 59.7)	95.6 (82.8, 98.2)	0.893 (0.824, 0.942)	<0.001
Prediabetes (all 125)	5.8	48.0 (38.3, 60.6)	72.6 (61.3, 81.9)	39.3 (28.7, 48.6)	73.8 (65.8, 82.1)	0.617 (0.556, 0.675)	<0.001
Disease							
PDAC (59)	5.8	44.1 (31.3, 50.6)	69.4 (57.2, 77.4)	45.6 (36.6, 55.8)	66.7 (57.4, 74.8)	0.55 (0.445, 0.652)	0.413
CP (35)	5.9	68.6 (57.1, 75.6)	70.7 (61.8, 79.4)	50.0 (38.2, 58.3)	72.7 (61.9, 82.2)	0.688(0.572, 0.790)	0.0027
PBLT (31)	5.6	64.5 (58.1, 77.4)	58.0 (49.3, 66.9)	28.3 (22.7, 36.8)	78.9 (71.4, 86.8)	0.610(0.507, 0.706)	0.0791

Values in table are presented as percentage with the 95% confidence interval in parenthesis.

AUC, area under the curve; PDAC, pancreatic ductal adenocarcinoma; CP, chronic pancreatitis; PBLT, pancreatic benign and low-grade tumors.

### 3.4 A “new combined” strategy

In our study, patients with pancreatic disease exhibited elevated levels of bilirubin, transaminase enzymes, aspartate transaminase, high density lipoprotein cholesterol and total bile acid (**Table 1**). Further analysis found that, for diabetes, FPG, HbA1c, 2h OGTT, total bilirubin, direct bilirubin, and alanine transaminase, the group diagnosed only by OGTT was higher in patients with pancreatic disease. (**Table 5**). Meanwhile, for prediabetes, 2h OGTT, lipase, alanine transaminase, total bile acid and total bilirubin in the group diagnosed by only the OGTT were higher in pancreatic diseases.

**Table 5:** Characteristic of participants identified as diabetes and prediabetes based on HbA1c and glucose in patients with pancreatic diseases.

	Diabetes			Prediabetes	
	By glucose not HbA1c(N=51)		By HbA1c not glucose(N=20)	By glucose not HbA1c(N=45)	By HbA1c not glucose(N=59)
Disease category (n, %)					
CP	9		9	9	18
PDAC	32		7	25	13
PBLT	10		4	11	28^***^
Age (year)	59.78 ± 12.03		54.30 ± 14.32	54.80 ± 13.58	52.41 ± 12.06
Male Sex (N, %)	32 (63)		12 (60)	24 (53)	39 (66)
Body mass index (kg/m^2^)	22.44 ± 3.57		22.21 ± 3.25	21.86 ± 2.91	22.35 ± 3.71
Systolic blood pressure (mmHg)	127.41 ± 18.31		125.15 ± 17.42	122.02 ± 16.10	120.97 ± 14.51
Diastolic blood pressure (mmHg)	80.14 ± 10.46		80.15 ± 10.05	78.44 ± 11.93	76.61 ± 10.41
Amylase (U/L)	91.35 ± 64.50		73.75 ± 57.34	132.73 ± 123.91	94.00 ± 76.43
HbA1c (%)	6.0 ± 0.4		7.0 ± 0.7^***^	5.2 ± 0.4	5.9 ± 0.0.2^***^
Fasting glucose (mmol/L)	6.24 ± 1.18		5.10 ± 1.15^***^	5.18 ± 0.68	5.11 ± 0.46
2-hour OGTT (mmol/L)	13.06 ± 2.70		8.36 ± 1.83^***^	8.73 ± 0.96	6.46 ± 0.79^***^
Total bilirubin (umol/L)	68.68 ± 95.44		14.03 ± 15.96^*^	75.18 ± 120.32	10.53 ± 3.34^***^
Alanine transaminase (IU/L)	61.14 ± 76.18		26.35 ± 23.22^*^	56.80 ± 61.80	21.78 ± 10.43^***^
Aspartate transaminase (IU/L)	68.46 ± 54.47		29.48 ± 19.21^*^	61.25 ± 53.37	25.49 ± 14.33^**^
Albumin (g/L)	42.10 ± 5.10		42.03 ± 3.29	41.86 ± 5.80	43.46 ± 3.65
Creatinine (umol/L)	66.31 ± 17.00		62.40 ± 18.70	63.62 ± 15.35	68.44 ± 14.94
Triglyceride (mmol/L)	1.70 ± 1.23		1.57 ± 0.82	1.52 ± 0.73	1.36 ± 0.83
Cholesterol (mmol/L)	4.59 ± 1.31		4.28 ± 2.03	4.57 ± 1.81	4.36 ± 0.76
High density lipoprotein cholesterol (mmol/L)	1.09 ± 0.71		1.11 ± 0.32	1.00 ± 0.52	1.23 ± 0.38^**^
Low density lipoprotein cholesterol (mmol/L)	2.27 ± 0.90		2.75 ± 1.27	2.49 ± 0.92	2.63 ± 0.66
Total bile acid (umol/L)	33.42 ± 57.62		10.56 ± 20.93	27.82 ± 61.56	5.23 ± 3.86^**^
Hemoglobin (g/L)	128.78 ± 18.88		129.75 ± 13.22	123.78 ± 18.85	133.94 ± 14.07^**^

Values are presented as mean ± SD. * P<0.05; ** P<0.01; *** P<0.001.

PDAC, pancreatic ductal adenocarcinoma; CP, chronic pancreatitis; PBLT, pancreatic benign and low-grade tumors.

The glucose criteria include the fasting plasma glucose or 2h OGTT.

**Figure 2:**
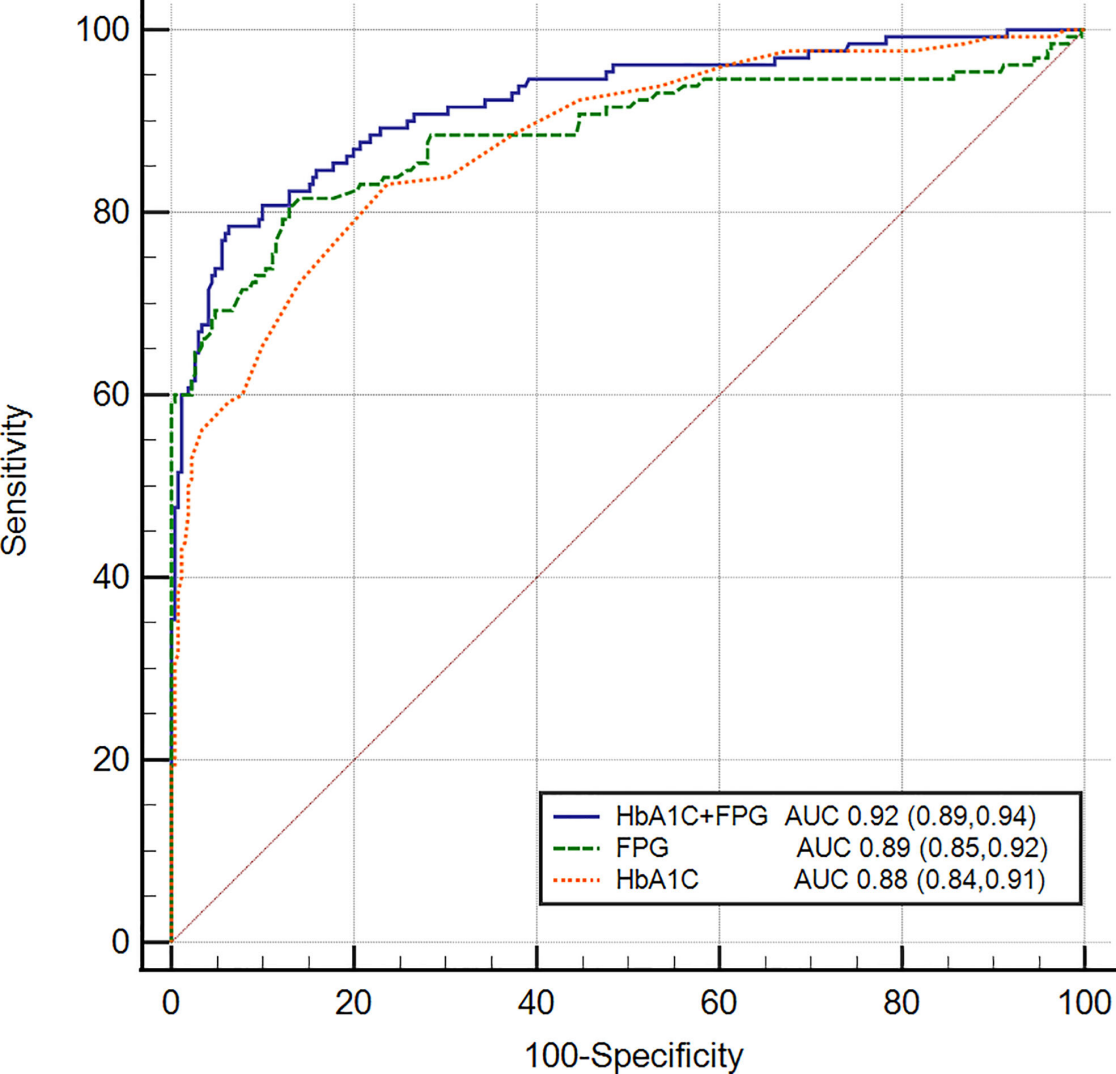
Receiver operating characteristic curves of FPG, HbA1c, and combined test for detecting pancreatic diabetes in all subjects. The AUC of the combined test was 0.919 (95% CI 0.888, 0.944) with the sensitivity of 85.7% and the specificity of 92.6%. Delong test was used to compared the AUC of combined test with FPG (P=0.012) and HbA1c (P=0.037).

In our study, glycated hemoglobin or FPG alone was not sufficient enough to diagnose diabetes correctly. We try to explore the performance of the combined tests (FPG>7.0 mmol/L or HbA1c >6.0%) for diagnosing diabetes in our patients. **Figure 2** shows the accuracy of the area under the value of HbA1c (6.0%), FPG (7.0 mmol/L) and the ‘new combined test’. The specificity was found to increase to 92.6% (95% CI 87.6, 97.3) when FPG and HbA1c test was used together, with a corresponding sensitivity of 85.7% (95% CI 79.1, 91.3), and the AUC was 0.919 (95% CI 0.888, 0.944). In our study, DeLong test showed that combined tests presented a higher accuracy compared with HbA1c (P=0.012) or FPG (P=0.037) alone, which demonstrated the new combined test may be a more efficient method to diagnose diabetes secondary to pancreatic disease.

## 4 Discussion

To assess the performance of HbA1c in detecting prediabetes or diabetes among patients with pancreatic disease, we analyzed the performance of HbA1c (6.5%) as recommended by the ADA criteria. The overall sensitivity and specificity of the HbA1c threshold (6.5%) for the diagnosis of diabetes in patients with pancreatic disease were 60.8% (95% CI: 52.3, 69.3) and 92.62% (95% CI: 89.5, 95.7), indicating that nearly 40% of diabetes cases went undiagnosed by HbA1c in our study. In prediabetes, similarly, HbA1c (5.7%) is not also a good predictor with low sensitivity and specificity of 53.2% (95%: CI 44.3, 62.0) and 59.6% (95% CI: 51.5, 67.6). Therefore, careful consideration is necessary when deciding whether to apply the ADA’s HbA1c criteria in this specific population.

In our study, the recommended glycated hemoglobin level by ADA is not sufficient for the diagnosis of diabetes or prediabetes in patients with pancreatic diseases. Thus, we analyzed the optimal threshold for prediabetes and diabetes through ROC curve. According to our data, the optimal cutoff value for diagnosing prediabetes was 5.8%, with the sensitivity and specificity of 48.0% (95% CI: 38.3, 60.6) and 72.6% (95% CI: 61.3, 81.9) respectively. The optimal cutoff value of 6.0% showed a higher sensitivity (83.8% vs 60.8%) and could detected more diabetes compared with the recommended value. In different pancreatic diseases, we obtained the optimal cutoff value respectively. These new cutoff values also showed a higher sensitivity with a subsequent improved detection rate.

The HbA1c threshold level of 6.5% for diagnosing T2DM has been widely discussed (8). Previous studies showed HbA1c is a reliable test that can be used both to diagnose and monitor diabetes (9). However, concerns about the utility of HbA1c for diagnosing DM and prediabetes have been recently raised (21, 22). Many studies have shown frequent discordance between the glucose and HbA1c criteria for diagnosing DM and prediabetes in different populations (11, 13, 23–26), which is consistent with our study. In patients with cystic fibrosis or liver cirrhosis, it is neither accurate nor reliable based on the A1C test (13, 27, 28). Tommerdahl et al. (27) found that screening for cystic fibrosis-related diabetes using an HbA1c cutoff point of 5.5% had a 78% sensitivity and 41% specificity, correlating to an ROC-AUC of 0.61. Sehrawat et al. (13) found that screening for diabetes in patients with cirrhosis using an HbA1c cutoff point of 6.5% had a 77.1% sensitivity and 90.8% specificity and noted that HbA1c was not sensitive enough to diagnose diabetes in patients with moderate to severe anemia.

Increasing data have shown that underlying factors, such as age, ethnics, diet, nutrition status, and specific disease, may affect the accuracy of HbA1c for diagnosing diabetes (8, 22, 29–31). We hypothesize that there may be various etiologies underlying pancreatic diseases. Firstly, abnormally elevated inflammatory factors, poor nutritional status and reduced enzyme activity was demonstrated in pancreatic disease, which may affect the production and clearance of red blood cells (32–34). Then, studies have demonstrated that individuals with diabetes due to pancreatic disease exhibit significant fluctuations in blood glucose levels (35). However, HBA1c is responsive to average fluctuations in blood glucose levels and may not be as sensitive to transient changes (36). Damage to the pancreatic duct is a common occurrence in pancreatic diseases which may lead to subsequent elevation of bilirubin and impaired liver function. Jaundice and reduced liver function, was the potential reasons for the impacting the HbA1c test. A study focusing on the associations between HbA1c and fatty liver markers revealed that alanine transaminase and aspartate transaminase increased with elevated HbA1c, implying that patients with high alanine transaminase or aspartate transaminase may be misdiagnosed as DM (37). A majority of studies have reported a negative correlation between bilirubin and glycosylated hemoglobin (17). Thus, Diagnostic testing for DM should be undertaken with caution due to the possibility that liver abnormalities could influence the predictive value of HbA1c and plasma glucose. In addition, the detection methods for HbA1c are of significant concern, as there exist various techniques. Hemoglobin variants can interfere with HbA1c methods for a variety of reasons (38). Presently, high-performance liquid chromatography by NGSP is the recommended method; however, its practicality in pancreatic diseases may be limited. Further research is necessary to identify tests that can alleviate the impact of pancreatic.

In our study, we found that it is not satisfactory to diagnose diabetes secondary to pancreatic disease when using either the conventional FPG criterion or HbA1c levels alone. Compared with HbA1c or FPG alone, the simultaneous measurement of HbA1c and FPG (HbA1c: 6.0% and FPG: 7.0 mmol/L) might be a tentative tool for identifying diabetes in patients with pancreatic diseases. Many studies have shown that, using a combination of an FPG of 7.0 mmol/L or higher and the HbA1c criterion improved diagnostic precision (39). In our study, using HbA1c with a value of 6.0% combined with FPG with a value of 7.0 mmol/L could increase the accuracy.

There were some limitations in our study. First, this was a cross sectional study, and we could not present evidence of a causal association between HbA1c levels and pancreatic diseases. Second, there was limited patients in our study, and a multicenter clinical trial evaluating the change in this specific population is essential. In addition, the diagnosis criteria for pancreatic diabetes is under controversial, and stringent standards need further be researched.

## 5 Conclusion

Based on the results of our study, we conclude that the recommended HbA1c of 5.7% and 6.5% by ADA criterion may not be sufficiently sensitive to diagnose prediabetes and diabetes secondary to pancreatic disease. The cutoff value of 5.8% and 6.0% improved the accuracy and should be considered to be applied. Besides, we advocate the combination of HbA1c (6.0%) and FPG (7.0 mmol/L) test for diagnosing diabetes secondary to pancreatic diseases.

## Data availability statement

The raw data supporting the conclusions of this article will be made available by the authors, without undue reservation.

## Ethics statement

The studies involving human participants were reviewed and approved by Medical Ethics Committee of West China Hospital at Sichuan University. The patients/participants provided their written informed consent to participate in this study. Written informed consent was obtained from the individual(s) for the publication of any potentially identifiable images or data included in this article.

## Author contributions

Study conception and design, acquisition of data, analysis and interpretation of data, writing the manuscript: GC, RZ, LY and YC and/or revision of the manuscript: All authors. All authors contributed to the article and approved the submitted version.

## References

[1] Association AD. Diagnosis and classification of diabetes mellitus. Diabetes Care (2014) 37(Suppl 1):S81–90. doi: 10.2337/dc14-S081 24357215

[2] Cui Y Andersen DK. Pancreatogenic diabetes: special considerations for management. Pancreatology Off J Int Assoc Pancreatology (2011) 11(3):279–94. doi: 10.1159/000329188 21757968

[3] Hart PA Bellin MD Andersen DK Bradley D Cruz-Monserrate Z Forsmark CE. Type 3c (pancreatogenic) diabetes mellitus secondary to chronic pancreatitis and pancreatic cancer. Lancet Gastroenterol Hepatol (2016) 1(3):226–37. doi: 10.1016/s2468-1253(16)30106-6 PMC5495015 28404095

[4] Wei Q Qi L Lin H Liu D Zhu X Dai Y. Pathological mechanisms in diabetes of the exocrine pancreas: what's known and what's to know. Front Physiol (2020) 11:570276. doi: 10.3389/fphys.2020.570276 33250773 PMC7673428

[5] Pannala R Leirness JB Bamlet WR Basu A Petersen GM Chari ST. Prevalence and clinical profile of pancreatic cancer-associated diabetes mellitus. Gastroenterology (2008) 134(4):981–7. doi: 10.1053/j.gastro.2008.01.039 PMC2323514 18395079

[6] Singhi AD Koay EJ Chari ST Maitra A. Early detection of pancreatic cancer: opportunities and challenges. Gastroenterology (2019) 156(7):2024–40. doi: 10.1053/j.gastro.2019.01.259 PMC6486851 30721664

[7] Goodarzi MO Petrov MS Andersen DK Hart PA. Diabetes in chronic pancreatitis: risk factors and natural history. Curr Opin Gastroenterol (2021) 37(5):526–31. doi: 10.1097/mog.0000000000000756 PMC8364494 34074860

[8] Zhou X Ruan X Hao L Zhou Y Gu J Qiu H. Optimal hemoglobin A1C cutoff value for diabetes mellitus and pre-diabetes in Pudong New Area, Shanghai, China. Prim Care Diabetes (2018) 12(3):238–44. doi: 10.1016/j.pcd.2017.12.006 29370998

[9] WHO Guidelines Approved by the Guidelines Review Committee. Use of glycated haemoglobin (HbA1c) in the diagnosis of diabetes mellitus: abbreviated report of a WHO consultation. Geneva: World Health Organization (2011). 26158184

[10] Association AD. Standards of medical care in diabetes–2013. Diabetes Care (2013) 36(Suppl 1):S11–66. doi: 10.2337/dc13-S011 PMC3537269 23264422

[11] Ren Q Lv X Yang L Yue J Luo Y Zhou L. Erythrocytosis and performance of HbA1c in detecting diabetes on an oxygen-deficient plateau: a population-based study. J Clin Endocrinol Metab (2020) 105(4). doi: 10.1210/clinem/dgaa001 31904080

[12] Nguyen KA Peer N de Villiers A Mukasa B Matsha TE Mills EJ. Glycated haemoglobin threshold for dysglycaemia screening, and application to metabolic syndrome diagnosis in HIV-infected africans. PLoS One (2019) 14(1):e0211483. doi: 10.1371/journal.pone.0211483 30703147 PMC6355005

[13] Sehrawat T Jindal A Kohli P Thour A Kaur J Sachdev A. Utility and limitations of glycated hemoglobin (HbA1c) in patients with liver cirrhosis as compared with oral glucose tolerance test for diagnosis of diabetes. Diabetes Ther (2018) 9(1):243–51. doi: 10.1007/s13300-017-0362-4 PMC5801248 29305791

[14] Edelson PK James KE Leong A Arenas J Cayford M Callahan MJ. Longitudinal changes in the relationship between hemoglobin A1c and glucose tolerance across pregnancy and postpartum. J Clin Endocrinol Metab (2020) 105(5):e1999–2007. doi: 10.1210/clinem/dgaa053 PMC7236626 32010954

[15] Chatzianagnostou K Vigna L Di Piazza S Tirelli AS Napolitano F Tomaino L. Low concordance between HbA1c and OGTT to diagnose prediabetes and diabetes in overweight or obesity. Clin Endocrinol (2019) 91(3):411–16. doi: 10.1111/cen.14043 31152677

[16] Nishida T. Diagnosis and clinical implications of diabetes in liver cirrhosis: a focus on the oral glucose tolerance test. J Endocr Soc (2017) 1(7):886–96. doi: 10.1210/js.2017-00183 PMC5686620 29264539

[17] Choi SW Lee YH Kweon SS Song HR Ahn HR Rhee JA. Association between total bilirubin and hemoglobin A1c in Korean type 2 diabetic patients. J Korean Med Sci (2012) 27(10):1196–201. doi: 10.3346/jkms.2012.27.10.1196 PMC3468756 23091317

[18] Kazemi A Ryul Shim S Jamali N Hassanzadeh-Rostami Z Soltani S Sasani N. Comparison of nutritional supplements for glycemic control in type 2 diabetes: a systematic review and network meta-analysis of randomized trials. Diabetes Res Clin Pract (2022) 191:110037. doi: 10.1016/j.diabres.2022.110037 35963372

[19] Chen SF Cui CL Wu P Xie NZ. Relationship of serum homocysteine level with nutritional status and HbA1c level in elderly inpatients. Int J Clin Exp Med (2013) 6(9):779–84. PMC3798213 24179571

[20] Zweig MH Campbell G. Receiver-operating characteristic (ROC) plots: a fundamental evaluation tool in clinical medicine. Clin Chem (1993) 39(4):561–77. 8472349

[21] Jørgensen ME Bjerregaard P Borch-Johnsen K Witte D. New diagnostic criteria for diabetes: is the change from glucose to HbA1c possible in all populations? J Clin Endocrinol Metab (2010) 95(11):E333–6. doi: 10.1210/jc.2010-0710 20739381

[22] Rhee M. HbA1c and diabetes: mismatches and misclassifications. J Clin Endocrinol Metab (2020) 105(7):e2630–2. doi: 10.1210/clinem/dgaa185 PMC7229987 32297930

[23] Li J Ma H Na L Jiang S Lv L Li G. Increased hemoglobin A1c threshold for prediabetes remarkably improving the agreement between A1c and oral glucose tolerance test criteria in obese population. J Clin Endocrinol Metab (2015) 100(5):1997–2005. doi: 10.1210/jc.2014-4139 25751104

[24] Eide IA Halden TA Hartmann A Åsberg A Dahle DO Reisæter AV. Limitations of hemoglobin A1c for the diagnosis of posttransplant diabetes mellitus. Transplantation (2015) 99(3):629–35. doi: 10.1097/tp.0000000000000376 25162478

[25] López López R Fuentes García R González-Villalpando ME González-Villalpando C. Diabetic by HbA1c, normal by OGTT: a frequent finding in the Mexico City diabetes study. J Endocrine Soc (2017) 1(10):1247–58. doi: 10.1210/js.2017-00266 PMC5686626 29264450

[26] Hsia DS Rasouli N Pittas AG Lary CW Peters A Lewis MR. Implications of the hemoglobin glycation index on the diagnosis of prediabetes and diabetes. J Clin Endocrinol Metab (2020) 105(3):e130–8. doi: 10.1210/clinem/dgaa029 PMC7015453 31965161

[27] Tommerdahl KL Brinton JT Vigers T Nadeau KJ Zeitler PS Chan CL. Screening for cystic fibrosis-related diabetes and prediabetes: evaluating 1,5-anhydroglucitol, fructosamine, glycated albumin, and hemoglobin A1c. Pediatr Diabetes (2019) 20(8):1080–86. doi: 10.1111/pedi.12914 PMC7585935 31469470

[28] Addepally NS George N Martinez-Macias R Garcia-Saenz-de-Sicilia M Kim WR Duarte-Rojo A. Hemoglobin A1c has suboptimal performance to diagnose and monitor diabetes mellitus in patients with cirrhosis. Digestive Dis Sci (2018) 63(12):3498–508. doi: 10.1007/s10620-018-5265-3 30159733

[29] Huang H Peng G Lin M Zhang K Wang Y Yang Y. The diagnostic threshold of HbA1c and impact of its use on diabetes prevalence-a population-based survey of 6898 han participants from southern China. Prev Med (2013) 57(4):345–50. doi: 10.1016/j.ypmed.2013.06.012 23777673

[30] Booth RA Jiang Y Morrison H Orpana H Rogers Van Katwyk S Lemieux C. Ethnic dependent differences in diagnostic accuracy of glycated hemoglobin (HbA1c) in Canadian adults. Diabetes Res Clin Pract (2018) 136:143–49. doi: 10.1016/j.diabres.2017.11.035 29203254

[31] Herman WH Cohen RM. Racial and ethnic differences in the relationship between HbA1c and blood glucose: implications for the diagnosis of diabetes. J Clin Endocrinol Metab (2012) 97(4):1067–72. doi: 10.1210/jc.2011-1894 PMC3319188 22238408

[32] Sato T Maekawa T Watanabe S Tsuji K Nakahata T. Erythroid progenitors differentiate and mature in response to endogenous erythropoietin. J Clin Invest (2000) 106(2):263–70. doi: 10.1172/jci9361 PMC314307 10903342

[33] Dzierzak E Philipsen S. Erythropoiesis: development and differentiation. Cold Spring Harbor Perspect Med (2013) 3(4):a011601. doi: 10.1101/cshperspect.a011601 PMC3684002 23545573

[34] Neri S Swinkels DW Matlung HL van Bruggen R. Novel concepts in red blood cell clearance. Curr Opin Hematol (2021) 28(6):438–44. doi: 10.1097/moh.0000000000000679 34494977

[35] Wynne K Devereaux B Dornhorst A. Diabetes of the exocrine pancreas. J Gastroenterol Hepatol (2019) 34(2):346–54. doi: 10.1111/jgh.14451 30151918

[36] Nitin S. HbA1c and factors other than diabetes mellitus affecting it. Singapore Med J (2010) 51(8):616–22. 20848057

[37] Katoh S Peltonen M Wada T Zeniya M Sakamoto Y Utsunomiya K. Fatty liver and serum cholinesterase are independently correlated with HbA1c levels: cross-sectional analysis of 5384 people. J Int Med Res (2014) 42(2):542–53. doi: 10.1177/0300060513517485 24595150

[38] Little RR Roberts WL. A review of variant hemoglobins interfering with hemoglobin A1c measurement. J Diabetes Sci Technol (2009) 3(3):446–51. doi: 10.1177/193229680900300307 PMC2769887 20144281

[39] Hu Y Liu W Chen Y Zhang M Wang L Zhou H. Combined use of fasting plasma glucose and glycated hemoglobin A1c in the screening of diabetes and impaired glucose tolerance. Acta diabetologica (2010) 47(3):231–6. doi: 10.1007/s00592-009-0143-2 19760291

