# Supplementary material for: Optimal glycated hemoglobin A1c value for prediabetes and diabetes in patients with pancreatic diseases
Source: Front Endocrinol (Lausanne). 2023 Jul 6;14:1208187. doi: 10.3389/fendo.2023.1208187 (PMC10358977; doi:10.3389/fendo.2023.1208187)
Supplement: Supplementary file 1 [file Table_1.docx]

| Supplement 1 Risk factors for diabetes by logistic regression analysis | | | | |
| --- | --- | --- | --- | --- |
|  | Univariate analysis | | Multivariate analysis | |
|  | OR (95% CI) | P | OR (95% CI) | p |
| Pancreatic disease |  |  |  |  |
| CP | 2.78 (1.46, 8.17) | 0.003 | 2.79 (1.66, 7.18) | 0.003 |
| PDAC | 4.08 (1.57, 7.48) | <0.0001 | 3.79 (1.991, 8.163) | <0.0001 |
| PBLT | 1.02 (0.79, 4.32) | 0.131 | Na |  |
| Age>55 | 2.48 (1.31, 3.88) | 0.035 | 1.81 (1.21, 3.54) | 0.060 |
| Male Sex (n, %) |  |  |  |  |
| female | ref |  |  |  |
| male | 1.15 (0.87, 3.57) | 0.663 | 1.12 (0.56, 2.18) | 0.717 |
| Body mass index (kg/m2) | 0.97 (0.66, 1.34) | 0.168 | NA |  |
| Systolic blood pressure (mmHg) | 0.79 (0.35, 1.02) | 0.137 | NA |  |
| Diastolic blood pressure (mmHg) | 1.04 (0.63, 3.88) | 0.153 | NA |  |
| Amylase (U/L) | 1.36 (1.01, 3.58) | 0.032 | NA |  |
| Total bilirubin (umol/L) | 1.07 (0.87, 1.69) | 0.056 | NA |  |
| Alanine transaminase (IU/L) | 1.01 (0.97, 1.04) | 0.863 | NA |  |
| Albumin (g/L) | 0.92 (0.81, 1.09) | 0.148 | NA |  |
| Creatinine (umol/L) | 1.13 (0.88, 1.27) | 0.472 | NA |  |
| Triglyceride (mmol/L) | 0.97 (0.63, 1.14) | 0.517 | NA |  |
| Cholesterol (mmol/L) | 0.97 (0.77, 1.05) | 0.833 | NA |  |
| High density lipoprotein cholesterol (mmol/L) | 0.52 (0.21, 0.96) | 0.016 | 0.47 (0.27, 0.89) | 0.035 |
| Low density lipoprotein cholesterol (mmol/L) | 0.95 (0.67, 1.13) | 0.372 | NA |  |
| Total bile acid (umol/L) | 1.00 (0.98, 1.02) | 0.416 | NA |  |
| Hemoglobin (g/L) | 1.01 (0.99, 1.23) | 0.075 | NA |  |
| PDAC, pancreatic ductal adenocarcinoma; CP, chronic pancreatitis; PBLT, pancreatic benign and low-grade tumors; OR, odds ratio; 95% CI, 95% confidence interval | | | | |
|  | | | | |
